# A newborn with seizures born to a mother diagnosed with primary carnitine deficiency

**DOI:** 10.1186/s12887-019-1452-4

**Published:** 2019-03-18

**Authors:** Si Chen, Yingying Hu, Yumei Huang, Yan Nan, Xiaojian Zhou, Shangqin Chen, Jin Lin, Zhenlang Lin

**Affiliations:** 1 Department of Neonatology, The Second Affiliated Hospital and Yuying Children’s Hospital of Wenzhou Medical University, Zhejiang, 325027 Wenzhou China; 2 Kravis Children’s Hospital and Department of Pediatrics, The Icahn School of Medicine at Mount Sinai, New York, NY 10029 USA

**Keywords:** Carnitine deficiency, Infant, Newborn, Amplitude integrated electroencephalogram, Magnetic resonance imaging, SLC22A5

## Abstract

**Background:**

Maternofetal carnitine transport through the placenta is the main route of fetal carnitine uptake. Decreased free carnitine levels discovered by newborn screening has identified many asymptomatic adult women with systemic primary carnitine deficiency (PCD). Here, we presented amplitude integrated electroencephalogram (aEEG) and magnetic resonance imaging (MRI) findings from a neonate with epilepsy whose mother was carnitine deficient.

**Case presentation:**

A one-day-old female newborn was admitted after experiencing seizures for half a day; status epilepticus was found on the continuous normal voltage background pattern with immature sleep-wake cycling during aEEG monitoring. On T1-weighted, T2-weighted, FLAIR, and DWI head MRI, there were various degrees of hyperintense signals and diffusion restrictions in the deep white matter of the right hemisphere. Tandem mass spectrometry discovered carnitine deficiency on the second day, which elevated to normal by the 9th day before L-carnitine supplementation was started. The patient was treated with phenobarbital after admission. No further seizures were noted by day 5. It was confirmed that the patient’s mother had a low level of serum-free carnitine. Gene analyses revealed that the newborn had heterozygote mutations on c.1400C > G of the SLC22A5 gene, and her mother had homozygous mutations on c.1400C > G. The patient had a good outcome at the 8-month follow up.

**Conclusions:**

Maternal carnitine deficiency that occurs during the perinatal period may manifest as secondary epilepsy with cerebral injury in neonates. The short-term neurodevelopmental outcomes were good. Early diagnosis of asymptomatic PCD in female patients can provide guidance for future pregnancies.

## Background

Carnitine (β-hydroxy-y-trimethylammonium butyrate) is a hydrophilic amino acid. Carnitine is an essential coenzyme for animals and plays an essential role in the transfer of long-chain fatty acids into mitochondria for subsequent β-oxidation. Carnitine deficiency can be primary or due to a number of acquired diseases, and it can ultimately impair fatty acid oxidization and lead to metabolic disorders. The clinical manifestations of carnitine deficiency encompass a broad spectrum and can vary widely according to the age of onset and involved organs [1]. The main clinical manifestations of carnitine deficiency are dilated cardiomyopathy, hepatomegaly and myasthenia [2]. Carnitine deficiency can also result in an acute metabolic decompensation with encephalopathy early in life, Reye-like syndrome [3] and sudden infant death [4]. Carnitine in the human body mainly comes from meat and dairy products, and a small amount is synthesized from lysine and methionine by liver and kidney cells. For fetuses, carnitine biosynthesis is immature; therefore, maternal-fetal carnitine transport through the placenta is the main route of fetal carnitine uptake, which plays an important role during fetal growth and development. After delivery, energy demands are dramatically increased for movement, growth, and differentiation as well as the maintenance of body temperature. All these processes depend on fatty acids oxidation facilitated by carnitine [5]. Here, we share a case of neonatal encephalopathy related to maternal primary carnitine deficiency.

## Case presentation

A one-day-old Han Chinese female infant was referred to our neonatal intensive care unit (NICU) after experiencing repeated seizures for half a day. She was the first-born child of a healthy, non-consanguineous Chinese couple. She was born at 40 weeks’ gestation via vaginal delivery. The birth weight was 3100 g. There was no intrauterine distress, birth trauma or asphyxia. There was no placental preface or abruption and no amniotic fluid pollution, and the umbilical cord was not wrapped around the neck. The Apgar scores were 10 and 10 at 1 min and 5 min after birth. Umbilical artery cord gas analysis was not done. She was breastfed after birth. A few hours after birth at the local hospital, the newborn suddenly experienced limb tremors with notable left side twitching after crying. The onset lasted for 3–4 min before subsiding on its own. Within half a day, a similar phenomenon occurred 6–7 times, with no observations of fever, tachypnea, screaming or vomiting. Upon her admission to the NICU at our facility, the infant appeared well and active. Her vital signs were normal: T37.3°C, P 119 bpm, RR 42 bpm, and BP 70/42 mmHg. The physical examination showed normal consciousness, good responses, and pink skin color. No jaundice was observed. The anterior fontanelle was flat and soft, with a size of approximately 1.0 × 1.0 cm. No cephalohematoma or bruising was present. The lungs were clear with equal aeration. Heart function was normal with no murmurs, and the abdomen was soft without hepatosplenomegaly. The parents did not have a similar disease, and no inherited metabolic diseases were known among family members.

**Fig. 1:**
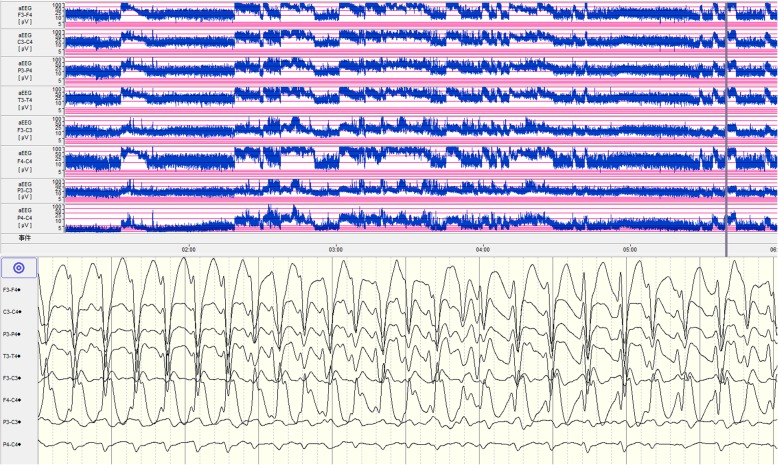
The aEEG of the 2-day-old patient. The aEEG showed a continuous normal voltage background pattern with immature sleep-wake cycling. Epileptic electrical activities in regular electroencephalogram at 10 s (the time corresponding with gray line in aEEG tracing) are displayed in the lower part of the figure. Epileptic electrical activities were mainly seen in right frontal temporal region

After admission, the patient was fed with formula milk and treated with phenobarbital followed by intravenous maintenance doses. Epileptic electrical activity was observed on a continuous normal voltage background pattern with immature sleep-wake cycling during amplitude integrated electroencephalogram(aEEG) monitoring (Fig. 1). Lab tests revealed normal serum glucose and high lactic acid levels (5.5 mmol/l), while ammonia levels were not tested. Blood gas tests revealed respiratory alkalosis, with PH 7.54, PCO_2_ of 20 mmHg, HCO_3_ of 21.5 mmHg, and BE of − 3.29 mmol/L. Electrolytes, liver enzymes, and creatine phosphokinase levels were normal. The newborn had no fever, vomiting, drowsiness or irritability. The physical examination indicated good responsiveness, a flat anterior fontanelle, normal muscle tone and no signs of nervous system dysfunction. Additionally, the complete blood count was normal: the leukocyte count was 12.3 × 10^9^/L with neutrophils prevailed, hemoglobin levels were 130 g/L, and platelet count was 371 × 10^9^/L. In addition, C-reactive protein was < 1.0 mg/L. There was no basis for intracranial infection; thus, we did not conduct a lumbar puncture. There were no obvious abnormal signs found in the head CT. Echocardiogram showed an atrial septal defect without cardiomyopathy.

**Fig. 2:**
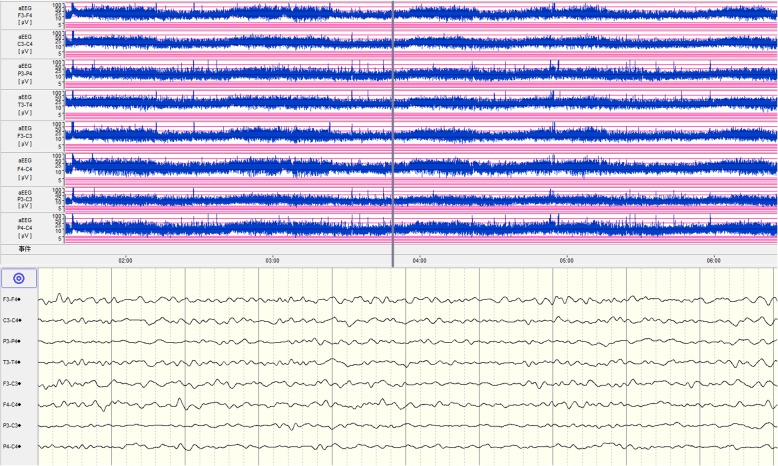
The aEEG of the 5-day-old patient. The aEEG showed a continuous normal voltage background pattern with mature sleep-wake cycling, and epileptic electrical activities had resolved. Primary electroencephalogram at 10 s (the time corresponding with gray line in aEEG tracing) are displayed in the lower part of the figure

**Fig. 3:**
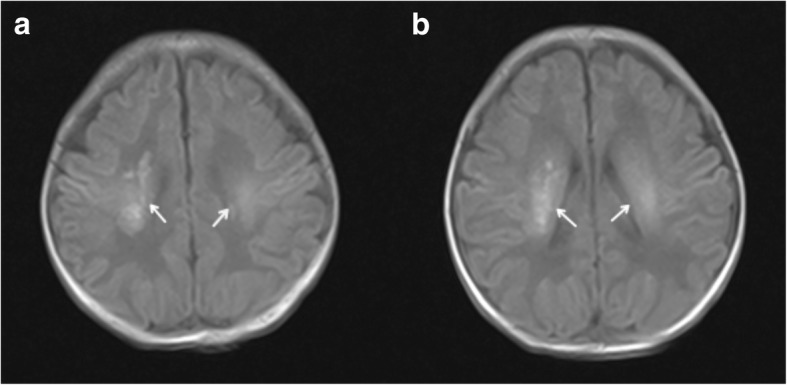
Head MRI of T1 WI axial images of life 5. **a** and **b** demonstrate asymmetric hyperintense changes in centrum semiovale and periventricular white matter, especially on the right side (see arrows)

On day 3 in the NICU, without additional anticonvulsants were administered, the convulsion frequency decreased. By day 5, no further clinical seizures were noted, and a repeated aEEG showed that epileptic electrical activities had resolved (Fig. 2). Phenobarbital was switched to oral treatment of 2.5 mg/kg twice a day. However, there were various degrees of hyperintense signals and diffusion restriction in the deep white matter of both hemispheres on T1-weighted (Fig. 3), T2-weighted, FLAIR, and DWI brain magnetic resonance imaging (MRI). On day 7, the blood bacteria culture was negative. On day 9, tandem mass spectrum of blood spots from day 2 revealed that free carnitine and several acyl carnitine levels had decreased (Table 1), and amino acid and organic acid levels were normal. The urinary tandem mass spectrum showed normal carnitine, amino acid, and organic acid levels. Decreased plasma free carnitine levels and several acyl carnitine levels suggested carnitine deficiency. The patient was treated with 150 mg/kg/d of L-carnitine intravenously on the following days. On day 13, the metabolic work up of the sample from day 9, just before L-carnitine treatment, indicated that plasma free carnitine and acyl carnitine levels increased to the normal lower limits (Table 1). The patient was discharged from the hospital on day 14. Considering brain lesions assessed by MRI and normal lower carnitine levels, the patient was given oral L-carnitine supplementation and phenobarbital after discharge. Half a month later, phenobarbital was discontinued.

**Table 1:** Plasma carnitine levels of the newborn

Carnitine species	From day 2/μmol/L	From day 9/μmol/L	Normal range/μmol/L
Free carnitine	8.65	12.38	9.50–60.00
C3 propionylcarnitine	0.36	0.47	0.40–5.00
C4 butyrylcarnitine	0.05	0.07	0.06–0.50
C4-OH-3-hydroxy butyrylcarnitine	0.02	0.06	0.04–0.50
C4DC succinyl carnitine	0.08	0.12	0.09–1.00
C14 myristoyl carnitine	0.05	0.04	0.06–0.45
C16 palmityl carnitine	0.39	0.55	0.45–6.00

**Fig. 4:**
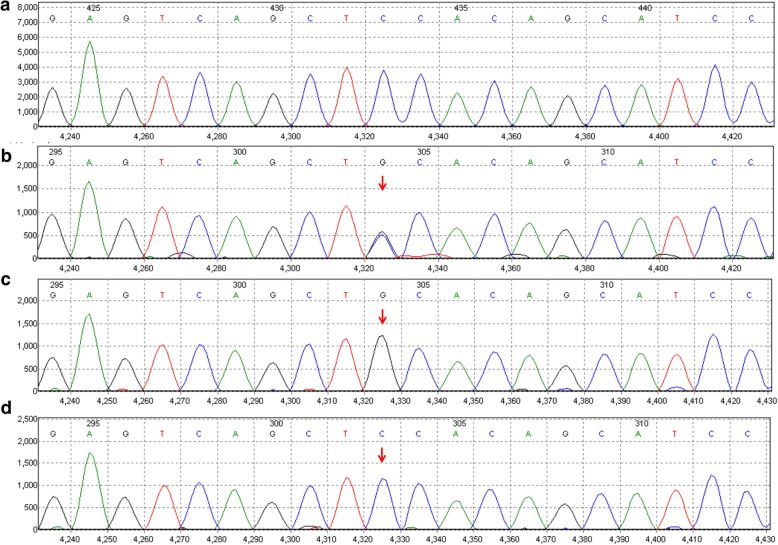
Mutation sites for c.1400C > G of the SLC22A5. **a** is for reference. The newborn had a heterozygote mutation for c.1400C > G of the SLC22A5 gene (**b**). Her mother had a homozygous mutation for c.1400C > G (**c**). Her father had normal SLC22A5 gene. The red arrows show mutation site (**d**)

Genetic testing showed that the patient had heterozygote mutations for c.1400C > G of the SLC22A5 gene. Her mother had homozygous mutations for c.1400C > G. Her father had a normal SLC22A5 gene (Fig. 4). It was confirmed that the patient’s mother had low plasma carnitine levels and was advised to start L-carnitine supplementation and to monitor carnitine levels at regular intervals, especially during future pregnancies.

**Fig. 5:**
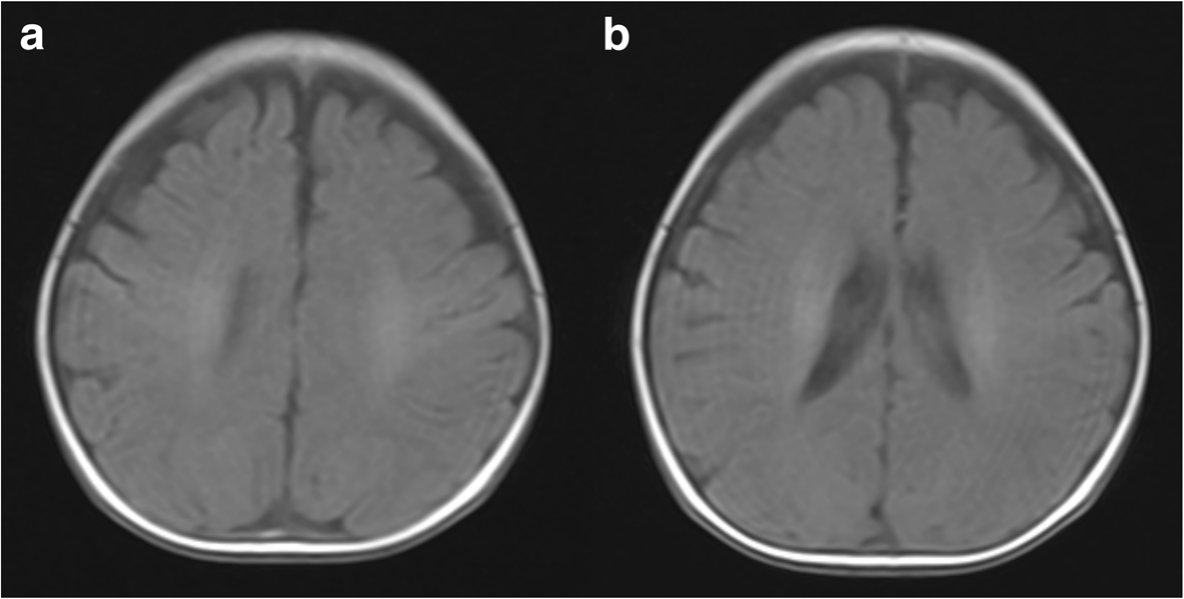
Head MRI of T1 WI axial images of life in the three-month-old. **a** and **b** demonstrate normal signals in centrum semiovale and periventricular white matter

When the patient was 3 months old, head MRI showed resolution of abnormal signals (Fig. 5). At more than 3 months of age, her repeat carnitine levels were elevated; thus, L-carnitine supplementation was decreased and then stopped 1 week later. At the 8-month follow up, levels of growth and development were assessed using the Gesell Developmental Scale [6], which yielded a developmental quotient (DQ) of 97.8, which is well within the normal range.

## Discussion

In this case, the clinical manifestations of seizure and head MRI were consistent with encephalopathy that resulted from carnitine deficiency. The differential diagnosis included intracranial infection, intracranial hemorrhage or mass, metabolic encephalopathy, bilirubin encephalopathy and hypoxic ischemic encephalopathy. Viral or bacterial central nervous system infection was excluded based on clinical presentation and physical examination. No risk factors for infection were observed, including chorioamnionitis, prolonged rupture of membranes, or fever, and the EEG was not typical of HSV encephalitis. Intracranial hemorrhage or mass was excluded since there were no obvious abnormal signs found on the head CT. Metabolic encephalopathy was excluded based on normal blood glucose and electrolyte levels. The infant had no hyperbilirubinemia and perinatal asphyxia; thus, bilirubin encephalopathy and hypoxic ischemic encephalopathy were also excluded.

After eliminating these potential causes for neonatal seizures, the diagnosis of carnitine deficiency was based on the observed low levels of plasma free carnitine and further confirmed by gene detection in the baby and mother [1]. In PCD patients, plasma free carnitine levels are usually below 5 μM. In this case, the newborn’s plasma free carnitine level was 8.65 μmol/L, and urinary carnitine levels were normal. The mother had a low plasma-free carnitine level due to homozygous mutations for c.1400C > G of SLC22A5. Therefore, the newborn was diagnosed with maternal carnitine deficiency.

It has been reported that carnitine deficiency impacts approximately 17% of patients with epilepsy [7]. Encephalopathy can be seen in secondary carnitine deficiency. Although it is unclear when encephalopathy occurs during the perinatal period, the rapid growth of the fetal brain and its high energy needs may explain the development of brain damage. The newborn’s plasma free carnitine level increased to 12.38 μmol/L on the ninth day after being fed with formula milk. Our formula milk contained 10 mg L-carnitine per 100 g powder (0.48 mg/100KJ). We continued L-carnitine therapy to further increase the baby’s carnitine level to a higher normal standard.

Brain damage can be seen on MRI imaging as hyperintense changes in the centrum semiovale and periventricular white matter [8]. The primary causes of encephalopathy are disorders in oxidative metabolism and metabolite accumulation caused by mitochondrial dysfunction, resulting in brain neuronal cell death, astrocyte myelin swelling and cerebral white matter damage [9]. Brain injury caused by secondary carnitine deficiency may be completely recovered after carnitine supplementation [9].

Primary carnitine deficiency (PCD) is an autosomal recessive disease caused by an SLC22A5 gene mutation that results in defective functional organic cation transporter 2 (OCTN2). PCD is characterized by increased losses in carnitine through the urine and decreased accumulation in serum and histocytes. In different countries or regions, the prevalence of PCD ranges from 1 in 300 to 1 in 120,000. In the Faroe Islands, the prevalence is 1 in 300 [10]; in the United States, it ranges from 1 in 20,000 to 1 in 70,000 [1], and, in China, it is 1 in 45,000 [11]. The incidence of heterozygotes in the population is between 0.5 and 1%. Newborn screening using tandem mass spectrometry can identify asymptomatic carriers and lead to the diagnosis of maternal PCD [12, 13]. On average, cells from asymptomatic women have higher levels of residual carnitine transport activity compared to symptomatic patients due to the presence of at least one missense mutation [14]. Among asymptomatic PCD female patients with the genotype, c.1400C > G (S467C) is one of the mutant genes. More specifically, a C.1400C > G gene mutation is located at the autosomal 5q31 with a missense mutation of an amino acid substitution from Ser to Cys at codon 467 (p. S467C) in exon 8 (reference sequence from NM_003060.3). This mutation was first reported by Koizumi in 1999 and is one of the most commonly mutated genes in patients with primary carnitine deficiency. There may be a relationship between plasma-free carnitine levels and OCTN2 genotypes; however, low plasma-free carnitine levels may not indicate disease severity or genotype [11]. The considerations described above may explain why the mother was asymptomatic. For these PCD patients, we recommend lifelong supplementation of L-carnitine, especially during future pregnancies, to prevent the occurrence of fetal carnitine deficiency.

## Conclusion

In summary, we report a case of a newborn with seizures who was born to a mother diagnosed with primary carnitine deficiency. Maternal carnitine deficiency that occurred during the perinatal period may have manifested as secondary epilepsy with cerebral injury in the neonate. Short-term neurodevelopmental outcomes were good, and no lifetime medication was required. Asymptomatic PCD women should begin L-carnitine supplementation and regularly monitor serum carnitine levels, especially during pregnancy, to prevent the occurrence of fetal carnitine deficiency.

## Abbreviations

aEEG: amplitude integrated electroencephalogram
MRI: magnetic resonance imaging
NICU: neonatal intensive care unit
PCD: primary carnitine deficiency
